# Variation in Management of Patients With Obstructive Coronary Artery Disease: Insights From the Veterans Affairs Clinical Assessment and Reporting Tool (VA CART) Program

**DOI:** 10.1161/JAHA.117.006336

**Published:** 2017-09-12

**Authors:** Amneet Sandhu, Maggie A. Stanislawski, Gary K. Grunwald, Kathryn Guinn, Javier Valle, Daniel Matlock, P. Michael Ho, Thomas M. Maddox, Steven M. Bradley

**Affiliations:** ^1^ Division of Cardiology University of Colorado School of Medicine Aurora CO; ^2^ Division of Cardiology VA Eastern Colorado Health Care System Aurora CO; ^3^ Minneapolis Heart Institute Minneapolis MN; ^4^ VA Eastern Colorado Health Care System University of Colorado School of Medicine Aurora CO; ^5^ Division of Geriatrics Department of Internal Medicine University of Colorado School of Medicine Aurora CO; ^6^ University of Colorado School of Medicine Aurora CO; ^7^ VA Eastern Colorado Geriatric Research Education and Clinical Center Denver CO; ^8^ Adult and Child Consortium for Outcomes Research and Delivery Science Aurora CO; ^9^ Department of Biostatistics and Informatics Colorado School of Public Health Aurora CO

**Keywords:** coronary artery bypass graft surgery, coronary artery disease, percutaneous coronary intervention, rate, variation, Coronary Artery Disease, Percutaneous Coronary Intervention, Cardiovascular Surgery

## Abstract

**Background:**

Little is known about facility‐level variation in the use of revascularization procedures for the management of stable obstructive coronary artery disease. Furthermore, it is unknown if variation in the use of coronary revascularization is associated with use of other cardiovascular procedures.

**Methods and Results:**

We evaluated all elective coronary angiograms performed in the Veterans Affairs system between September 1, 2007, and December 31, 2011, using the Clinical Assessment and Reporting Tool and identified patients with obstructive coronary artery disease. Patients were considered managed with revascularization if they received percutaneous coronary intervention (PCI) or coronary artery bypass grafting within 30 days of diagnosis. We calculated risk‐adjusted facility‐level rates of overall revascularization, PCI, and coronary artery bypass grafting. In addition, we determined the association between facility‐level rates of revascularization and post‐PCI stress testing. Among 15 650 patients at 51 Veterans Affairs sites who met inclusion criteria, the median rate of revascularization was 59.6% (interquartile range, 55.7%–66.7%). Across all facilities, risk‐adjusted rates of overall revascularization varied from 41.5% to 88.1%, rate of PCI varied from 23.2% to 80.6%, and rate of coronary artery bypass graftingvariedfrom 7.5% to 36.5%. Of 6179 patients who underwent elective PCI, the median rate of stress testing in the 2 years after PCI was 33.7% (interquartile range, 30.7%–47.1%). There was no evidence of correlation between facility‐level rate of revascularization and follow‐up stress testing.

**Conclusions:**

Within the Veterans Affairs system, we observed large facility‐level variation in rates of revascularization for obstructive coronary artery disease, with variation driven primarily by PCI. There was no association between facility‐level use of revascularization and follow‐up stress testing, suggesting use rates are specific to a particular procedure and not a marker of overall facility‐level use.

**Clinical Perspective**

**What Is New?**

- Across the largest integrated health network in the United States, we describe large facility‐level variation in rates of revascularization for obstructive coronary artery disease and use of stress testing following revascularization.
- In addition, we found patterns of use for a specific procedure may not be a marker of facility‐level use of other procedures.

**What Are the Clinical Implications?**

- This work found variation in the approach to obstructive coronary artery disease in the absence of financial drivers, highlighting the importance of understanding factors driving this variability (eg, local culture, process of care, and resource availability) that may inform opportunities to achieve more consistent effective care.

Strategies for revascularization of obstructive coronary artery disease (CAD), such as percutaneous coronary intervention (PCI) or coronary artery bypass grafting (CABG), are invasive and costly treatment options. In the setting of acute myocardial infarction, revascularization reduces the risk of mortality and future myocardial infarction. In contrast, the benefits of revascularization for stable CAD provide no mortality advantage over management with optimal medical therapy. Accordingly, targeted use of revascularization is important to optimize patient outcomes without adding unnecessary cost.

Prior studies have consistently shown significant regional variation in the overall use of revascularization procedures.^1^, ^2^, ^3^ However, less is known about the use of revascularization procedures within integrated healthcare systems, like the Veterans Affairs (VA) system. Furthermore, studies evaluating variation in the use of revascularization or medical therapy in patients with newly diagnosed stable CAD are lacking.

Although procedural use patterns may not track across clinical conditions, this remains to be explored within a disease state such as CAD. In addition, studies of variation in care use are often limited to the study of a single procedure or procedure type. Evaluating variation in the rates of revascularization procedures relative to other cardiovascular procedures may provide insights into a site's overall use pattern. For example, if sites with high rates of revascularization also perform post‐PCI stresstesting more frequently during follow‐up, understanding site‐level rates of revascularization might serve as a marker of overall use of cardiovascular procedures. If rates of revascularization procedures are unrelated to follow‐up stress testing, this would suggest use metrics need to be granular as an overall fingerprint of use cannot be informed by 1 type of procedure.

In this study, we sought to describe variation in the use of revascularization procedures for patients with newly diagnosed, stable, obstructive CAD by elective coronary angiography in the VA system. In addition, we sought to determine if variation in revascularization was predominantly related to differences in use of PCI or CABG. Finally, we evaluated the association between facility‐level rates of revascularization and use of stress testing in the 2 years following PCI as stress testing within 2 years of PCI is rarely appropriate in the absence of recurrent or progressive symptoms. Findings of this study may inform future approaches to address variation in use of revascularization procedures and the extent to which single measures of use reflect larger use patterns across a clinical condition.

## Methods

### Data Source

Data for this study were derived from the VA Clinical Assessment, Reporting Tool (CART).^4^ CART is a national VA quality improvement program designed for all VA catheterization laboratories. The program uses a clinical software application that is embedded in the VA electronic health record to collect standardized patient and procedural variables for all coronary procedures performed at all VA catheterization laboratories nationally. CART data elements mirror those from the American College of Cardiology National Cardiovascular Data Repository data definitions. Longitudinal patient data are captured by combining CART data with information from the VA patient electronic health record, including clinic visits, laboratory analysis, medications and prescriptions, inpatient hospitalizations, and vital status. This is further merged with VA fee‐based data to account for non‐VA care and hospitalizations paid for by the VA. CART internal quality checks are periodically conducted for completeness and accuracy. Data validity in the CART system has been demonstrated in prior studies.^5^ Institutional review board and VA research and development approvals were obtained for the analysis conducted in this study, with approval for waiver of patient consent.

### Study Population

#### Stable obstructive CAD

**Figure 1:**
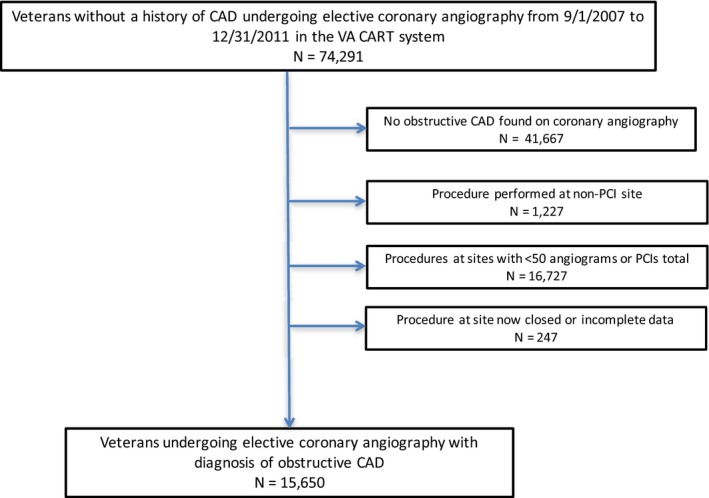
Cohort design showing inclusion and exclusion criteria. CAD indicates coronary artery disease; CART, Clinical Assessment and Reporting Tool; PCI, percutaneous coronary intervention; and VA, Veterans Affairs.

We identified 74 291 patients without a known history of CAD undergoing elective coronary angiography at the VA through the VA CART system between September 1, 2007, and December 31, 2011. We excluded 41 667 patients with nonobstructive or no CAD on angiography (defined as <50% angiographic stenosis of the left main coronary artery or <70% angiographic stenosis of any major epicardial coronary vessel) and 247 patients with incomplete angiographic data. To avoid inflating variance related to facilities with small numbers or lacking access to revascularization, we excluded 17 954 patients from VA facilities without PCI capability or low‐volumes VA sites (<50 angiograms or PCIs total during the study period). Figure 1 shows an attrition plot deriving our study cohort that included 15 650 patients at 51 VA sites with a new or initial diagnosis of obstructive CAD.

#### Stress testing after PCI

Outside of new or progressive symptoms, stress testing within 2 years of PCI is rarely appropriate.^6^ In light of this criterion, we have previously evaluated site‐level variation in the use of stress testing after PCI.^7^ What remains unknown is the site‐level relationship between PCI for obstructive CAD and use of stress testing after PCI. To evaluate for a possible relationship, we identified 6179 veterans undergoing elective PCI, excluding those with a missing indication for PCI, known prior CAD, a nonelective indication for procedure, death within 60 days of PCI, and/or a procedure at low‐volume PCI facilities in the same study period.

Use of stress testing in the 2 years after PCI was identified from the VA Corporate Data Warehouse using Current Procedural Terminology and *International Classification of Disease, Ninth Revision*, codes for stress echocardiography. Electrocardiographic or pharmacological stress and nuclear imaging procedures performed within 72 hours of each other were considered a single test event. Similarly, pharmacological stress and echocardiographic or magnetic resonance imaging testing performed on the same day were considered to be a single test. Per prior studies,^8^, ^9^ a 60‐day blackout period after PCI was imposed to allow for stress testing for purposes of procedure staging, cardiac rehabilitation, or assessment of functional capacity.^7^

### Outcome Variables

The outcome of interest for the cohort of patients with newly diagnosed, stable, obstructive CAD was any revascularization, either PCI or CABG, within 30 days of angiographic diagnosis of obstructive CAD. In the cohort of patients with PCI, the outcome of interest was stress testing within 2 years of follow‐up. We linked VA CART data to Medicare data, allowing us to capture revascularization events and stress tests that may have been conducted outside of the VA system.

### Statistical Analysis

Comparisons of patient and procedural characteristics by hospital quartile of revascularization rates were completed using the Kruskall–Wallis test for continuous variables and the χ^2^ or Fisher exact test for categorical variables. Among patients with stable CAD, we determined hospital‐level variation in risk‐standardized rates of revascularization, PCI, and CABG within 30 days of elective diagnostic coronary angiogram using mixed logistic regression models. All models included hospital random intercepts to account for clustering of patients within hospitals and were adjusted for the following patient‐level covariates: demographics (age, sex, and white race), clinical risk factors (diabete smellitus, tobacco use, hypertension, hyperlipidemia, peripheral arterial disease, cerebral vascular disease, congestive heart failure, obese or overweight, chronic obstructive pulmonary disease, and chronic kidney disease), results of coronary angiogram (coronary disease severity: 1, 2, or 3 vessels or left main; and obstructive disease in proximal left anterior descending [LAD]), and year of procedure. All of these covariates were chosen a priori on the basis of clinical judgment and previously published studies. Models were estimated using Bayesian Markov chain Monte Carlo methods.^10^ These methods provide shrinkage estimators and precision intervals for hospital‐specific revascularization rates that adjust for patient characteristics and account for differences in hospital sample size. The methods also provide an estimate of median odds ratio (MOR) to further quantify variation in revascularization across hospitals.^11^, ^12^

Among patients who underwent PCI, we calculated similar measures of facility‐level variation and rates of stress testing in the 2 years after PCI using the same Bayesian methods, but additionally controlling for stent type (drug‐eluting or bare metal stent) and PCI indication. To assess for potential correlation, we then compared Markov chain Monte Carlo estimated facility rates of revascularization for obstructive CAD to estimated rates of stress testing in the 2 years after PCI using Pearson's correlation.

In addition, we performed a sensitivity analysis among sites with on‐site cardiac surgery (70% of sites). We used the same statistical models as described above to estimate risk‐adjusted rates of 30‐day revascularization, PCI, and CABG, as well as stress testing after PCI. We cross‐referenced CART data with VA patient data files. Most variables had no missing values. Exceptions included race (missing, <10%), and height or weight (missing, <1%), which were used to calculate body mass index and, hence, overweight/obese. Statistical analyses were performed with SAS v9.4 and R v3.3.1, and all statistical tests were evaluated at a significance level of 0.05.

## Results

Among 15 650 patients with a new diagnosis of obstructive stable CAD in the VA system, 9455 (60.4%) underwent revascularization within 30 days, with 6090 (64.4%) receiving PCI and 3365 (35.6%) receiving CABG. Table 1 displays patient and hospital characteristics by hospital quartiles of median facility‐level rates of overall revascularization within 30 days of angiographic diagnosis of obstructive coronary disease. Although statistically significant because of sample size, no clinically significant differences were noted in patient demographics or comorbidities between hospital quartiles. Similarly, there were no clinically significant differences for indication of elective coronary angiography between hospital quartiles. The rate of proximal LAD, 3‐vessel, or left main obstructive coronary disease was seen to be higher in quartile 4 (highest rate of revascularization in the 30 days after diagnosis of obstructive CAD) compared with quartiles 1 to 3. Otherwise, there were no clinically significant differences in the rates of obstructive CAD between groups.

**Table 1:** Patient Demographics, Comorbidities, Medications, Angiographic Findings, Treatment Strategy, and Facility Characteristics by Hospital Quartile Based on Rate of Revascularization for Obstructive CAD

Variable	Total (N=15 650)	Quartile 1 (n=4309)	Quartile 2 (n=3832)	Quartile 3 (n=3844)	Quartile 4 (n=3665)	*P* Value
Demographics						
Age, median (IQR), y	63.7 (60.0–69.5)	63.9 (60.1–70.1)	63.6 (59.8–69.0)	63.7 (60.0–69.7)	63.7 (59.9–69.1)	0.016
Sex, male	15 417 (98.5)	4249 (98.6)	3773 (98.5)	3789 (98.6)	3606 (98.4)	0.85
White race	12 539 (80.1)	3442 (79.9)	2922 (76.3)	3196 (83.1)	2979 (81.3)	<0.0001
Risk factors and comorbidities						
Tobacco use	8674 (55.4)	2337 (54.2)	2043 (53.3)	2195 (57.1)	2099 (57.3)	0.0003
Diabetes Mellitus	6923 (44.2)	1889 (43.8)	1723 (45.0)	1704 (44.3)	1607 (43.8)	0.72
Hypertension	13 443 (85.9)	3744 (86.9)	3236 (84.4)	3281 (85.4)	3182 (86.8)	0.0034
Hyperlipidemia	13 300 (85.0)	3693 (85.7)	3247 (84.7)	3225 (83.9)	3135 (85.5)	0.094
Cholesterol, mg/dL median (IQR)	172.0 (148.3–200.0)	170.5 (147.3–198.3)	174.0 (149.0–202.0)	171.1 (147.5–199.0)	174.0 (150.0–202.0)	0.0002
LDL, mg/dL median (IQR)	99.3 (79.3–124.3)	98.5 (78.0–124.0)	100.0 (79.7–125.3)	99.9 (80.0–124.0)	99.3 (80.0–124.1)	0.13
HDL, mg/dL median (IQR)	37.8 (32.0–45.0)	38.5 (32.7–46.0)	38.0 (32.5–45.0)	36.0 (31.0–43.0)	38.0 (32.2–45.0)	<0.0001
Peripheral arterial disease	2799 (17.9)	781 (18.1)	702 (18.3)	622 (16.2)	694 (18.9)	0.012
Cerebrovascular disease	2112 (13.5)	595 (13.8)	507 (13.2)	499 (13.0)	511 (13.9)	0.55
Congestive heart failure	1685 (10.8)	468 (10.9)	445 (11.6)	390 (10.1)	382 (10.4)	0.18
Chronic obstructive pulmonary disease	2603 (16.6)	713 (16.5)	620 (16.2)	671 (17.5)	599 (16.3)	0.44
Chronic kidney disease	2193 (14.0)	621 (14.4)	571 (14.9)	515 (13.4)	486 (13.3)	0.11
Dialysis	310 (2.0)	76 (1.8)	84 (2.2)	59 (1.5)	91 (2.5)	0.014
GFR, ml/min median (IQR)	75.5 (60.2–90.0)	76.3 (61.6–90.3)	76.0 (62.0–90.4)	76.0 (63.0–90.2)	72.4 (60.0–88.7)	<0.0001
Obese	7513 (58.8)	2055 (56.5)	1874 (58.2)	1879 (60.2)	1705 (60.6)	0.002
Overweight	5683 (47.5)	1562 (45.3)	1397 (46.0)	1370 (47.7)	1354 (52.0)	<0.0001
BMI, kg/m^2^ median (IQR)	29.8 (26.5–33.6)	29.7 (26.3–33.4)	29.9 (26.6–34.0)	29.8 (26.5–33.7)	29.6 (26.3–33.3)	0.0084
Alcohol abuse	1182 (7.6)	329 (7.6)	292 (7.6)	289 (7.5)	272 (7.4)	0.98
Substance abuse/dependence	500 (3.2)	137 (3.2)	123 (3.2)	122 (3.2)	118 (3.2)	>0.99
Chronic depression	3805 (24.3)	985 (22.9)	893 (23.3)	1008 (26.2)	919 (25.1)	0.0012
Framingham risk category						
High	5778 (36.9)	1440 (33.4)	1288 (33.6)	1447 (37.6)	1603 (43.7)	<0.0001
Medium	7567 (48.4)	2224 (51.6)	1956 (51.0)	1744 (45.4)	1643 (44.8)	
Low	2305 (14.7)	645 (15.0)	588 (15.3)	653 (17.0)	419 (11.4)	
Procedural indication						
Stable angina	645 (4.1)	118 (2.7)	165 (4.3)	204 (5.3)	158 (4.3)	<0.0001
Chest pain	8642 (55.2)	2408 (55.9)	1981 (51.7)	2202 (57.3)	2051 (56.0)	
Dysrhythmia	75 (0.5)	24 (0.6)	15 (0.4)	10 (0.3)	26 (0.7)	
Ischemic heart disease	1124 (7.2)	277 (6.4)	315 (8.2)	235 (6.1)	297 (8.1)	
Positive functional study	3370 (21.5)	932 (21.6)	886 (23.1)	775 (20.2)	777 (21.2)	
Missing/unknown	1794 (11.5)	550 (12.8)	470 (12.3)	418 (10.9)	356 (9.7)	
Prior stress test	13 991 (89.4)	3833 (89.0)	3470 (90.6)	3416 (88.9)	3272 (89.3)	0.058
Preprocedural medication						
Statins	10 035 (64.1)	2780 (64.5)	2413 (63.0)	2494 (64.9)	2348 (64.1)	0.32
βBlockers	9083 (58.0)	2592 (60.2)	2089 (54.5)	2267 (59.0)	2135 (58.3)	<0.0001
Calcium channel blockers	3986 (25.5)	1116 (25.9)	981 (25.6)	901 (23.4)	988 (27.0)	0.0045
Nitrates	6463 (41.3)	1891 (43.9)	1430 (37.3)	1608 (41.8)	1534 (41.9)	<0.0001
Coronary summary						
1V obstructive	6779 (43.3)	1974 (45.8)	1594 (41.6)	1687 (43.9)	1524 (41.6)	0.0014
2V obstructive	4057 (25.9)	1080 (25.1)	1030 (26.9)	989 (25.7)	958 (26.1)	
3V or left main obstructive	4814 (30.8)	1255 (29.1)	1208 (31.5)	1168 (30.4)	1183 (32.3)	
Proximal LAD obstructive disease	3165 (20.2)	828 (19.2)	721 (18.8)	766 (19.9)	850 (23.2)	<0.0001
Treatment						
CABG	3365 (21.5)	778 (18.1)	857 (22.4)	849 (22.1)	881 (24.0)	<0.0001
Medical therapy only	6195 (39.6)	2145 (49.8)	1613 (42.1)	1448 (37.7)	989 (27.0)	
PCI	6090 (38.9)	1386 (32.2)	1362 (35.5)	1547 (40.2)	1795 (49.0)	
Treatment facility						
Cardiac surgery on site	11 034 (70.5)	2960 (68.7)	2657 (69.3)	2646 (68.8)	2771 (75.6)	<0.0001

Data are number (percentage) unless otherwise indicated. 1V,1 vessel; 2V, 2 vessels; 3V, 3 vessels; BMI, body mass index; CABG, coronary artery bypass grafting; CAD, coronary artery disease; GFR, glomerular filtration rate; HDL, high‐density lipoprotein; IQR, interquartile range; LAD, left anterior descending; LDL, low‐density lipoprotein; PCI, percutaneous coronary intervention.

**Figure 2:**
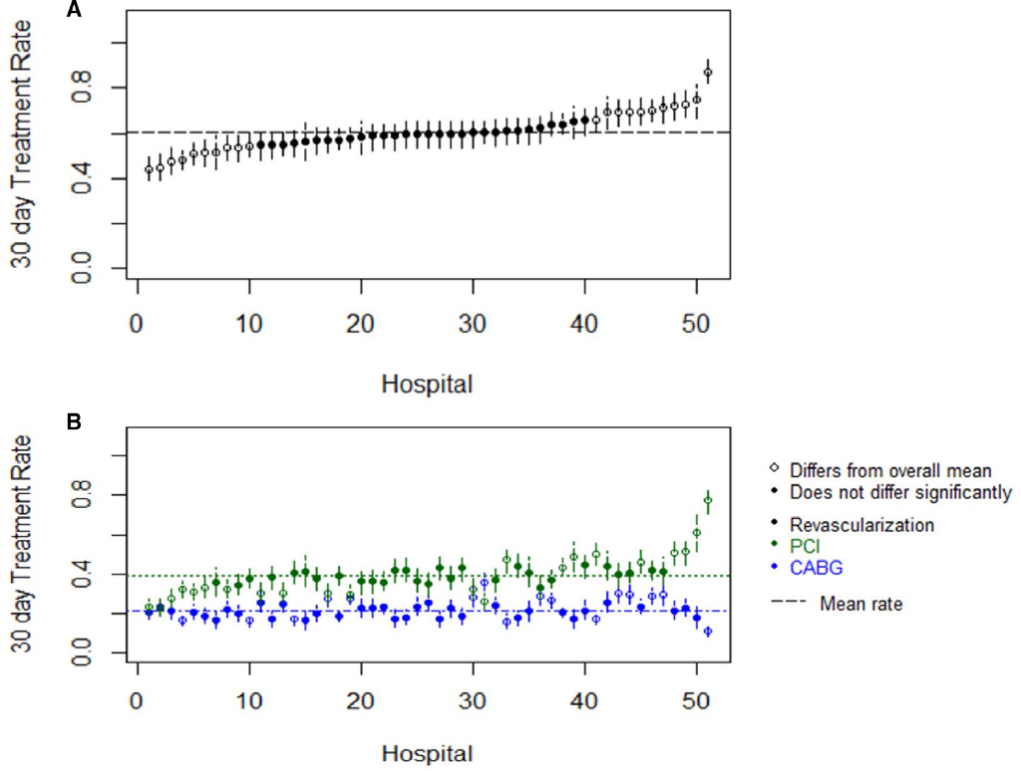
A, Total risk‐adjusted rate of revascularization of obstructive coronary artery disease (CAD) across all hospitals. B, Risk‐adjusted rate of revascularization by percutaneous coronary intervention (PCI) and coronary artery bypass grafting (CABG).

Table 2 shows the median rates of total revascularization, PCI, and CABG by hospital quartile based on rate of revascularization of obstructive CAD. In the 30 days after diagnosis of obstructive CAD by elective coronary angiography, the overall risk‐standardized facility‐level median revascularization rate was 59.6% for any revascularization procedure (range, 41.5%–88.1%), 39.6% for PCI (range, 23.3%–80.6%), and 20.2% for CABG (range, 7.5%–36.5%). Figure 2A and 2B display the rates of risk‐adjusted revascularization across all hospitals included in this study as well as revascularization by PCI or CABG, respectively. In a sensitivity analysis analyzing 36 VA sites with on‐site cardiothoracic surgery services available, no significant rate for overall revascularization (59.0%; range, 45.1%–73.2%), PCI (37.8%; range, 23.3%–58.6%), and CABG (23.6%; range, 17.3%–36.1%) was noted compared with analysis of all facilities.

**Table 2:** Median Rates of Total Revascularization, PCI, and CABG by Hospital Quartile Based on Rate of Revascularization of Obstructive CAD

Treatment	Hospital Quartile 1	Hospital Quartile 2	Hospital Quartile 3	Hospital Quartile 4	All
Revascularization	52.1 (48.5–53.3; 41.5–55.7)	57.8 (56.7–58.8; 56.1–59.6)	61.5 (60.2–62.5; 59.6–66.7)	72.2 (69.8–75.1; 66.9–88.1)	59.6 (55.7–66.7; 41.5–88.1)
PCI	32.9 (29.9–35.5; 23.3–41.2)	37.2 (34.6–39.9; 27.1–43.1)	44 (35.1–45.2; 25–52.8)	45.1 (40.7–53.6; 37.3–80.6)	39.6 (34.2–44; 23.3–80.6)
CABG	18.2 (16.6–20.2; 13.5–24.5)	20.6 (17.7–23.1; 14.9–29.5)	20 (16.2–26.3; 13.3–36.5)	27 (19.9–30.3; 7.5–31.9)	20.2 (16.6–26.3; 7.5–36.5)

Data are given as median (interquartile range; range). CABG, coronary artery bypass grafting; CAD, coronary artery disease; PCI, percutaneous coronary intervention.

In addition, we used the MOR to quantify the extent to which variation in rates of revascularization was explained by differences across hospitals. The MOR can be interpreted as the odds that 2 similar patient‐level covariates from separate randomly chosen hospitals will receive revascularization after diagnosis of obstructive CAD at elective angiography. The MOR was 1.48 for total revascularization, 1.60 for PCI, and 1.48 for CABG, implying 60% greater odds of PCI and 48% greater odds of CABG for patients with similar covariates receiving either procedure at 1 randomly selected VA hospital compared with another. To evaluate for a potential difference in rate of revascularization for disease states with an agreed on treatment strategy, we analyzed the subset of patients (n=9660) with an elective diagnosis of obstructive multivessel CAD, proximal LAD, or left middle cerebral artery disease. The MOR for this subset was 1.5 (1.38–1.67 ).

**Figure 3:**
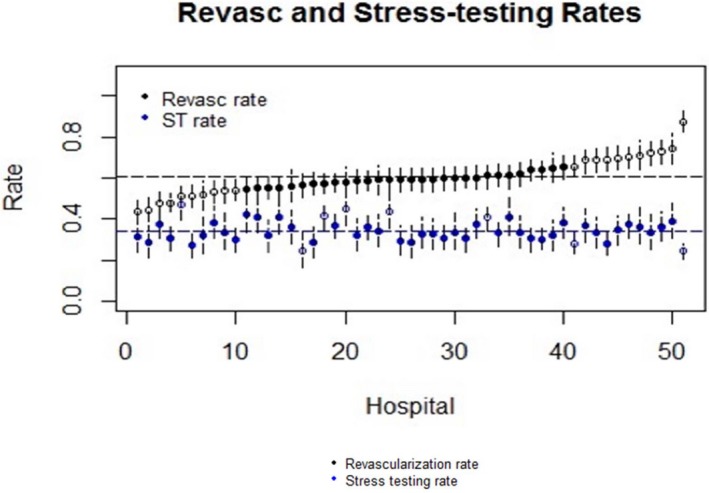
Risk‐adjusted rate of revascularization compared with 2‐year post–percutaneous coronary intervention stress testing rate by hospital.

Of the 6179 patients who underwent elective PCI during the study period, 2111 (34.2%) underwent stress testing within 2 years after PCI. The median risk‐adjusted facility‐level rate of stress testing in the 2 years after PCI was 33.7% (interquartile range, 30.7%–47.1%). Figure 3 displays the comparison of revascularization rates in the 30 days after identification of obstructive CAD and 2‐year stress testing rates after PCI by hospital. There was no significant correlation between site‐level revascularization of obstructive CAD and the 2‐year stress test rate after elective PCI (correlation coefficient,−0.09; *P*=0.49).

## Discussion

In this study, we sought to describe facility‐level variation in the use of revascularization for the initial management of newly diagnosed, stable, obstructive CAD by elective coronary angiography performed in the VA system. In addition, we determined the association between facility‐level rates of revascularization and stress testing in follow‐up of PCI. After diagnosis of obstructive CAD, the median facility‐level rate of revascularization was 59.6%, with large variability between hospitals, from 41.5% to 88.1%, largely driven by the rate of PCI. We found no correlation between site‐level rate of revascularization for obstructive CAD and 2‐year rate of stress testing after elective PCI. These findings show large facility‐level variation in 2 procedures, rates of revascularization for obstructive CAD and use of stress test following, and suggest patterns of use for a specific procedure may not be a marker of facility‐level use of other procedures.

Many studies have shown regional variation in the use of diagnostic coronary angiography and coronary revascularization procedures.^13^, ^14^, ^15^ In these prior studies, variation may have been driven by differences in the rate of obstructive CAD in populations rather than use of revascularization procedures. Furthermore, studies of variation in the rates of PCI relative to CABG among patients undergoing revascularization have often lacked detail on the angiographic findings that may influence use of CABG (ie, left main or 3‐vessel CAD).^16^ What has remained unclear is whether variation in revascularization persists when patient populations are restricted to those eligible for the procedure on a new diagnosis of obstructive CAD by elective coronary angiography. The present study directly addresses these prior limitations and suggests persistent variation even among restricted populations and after accounting for patient and procedural characteristics.

Although there is a paucity of data describing initial treatment approaches for stable obstructive CAD, a study from Canada suggests nearly 2 of 3 patients undergo revascularization within 90 days of diagnosis by angiography.^17^ Limited prior work describing facility rates of PCI to CABG has also shown determinants of variation in coronary revascularization practices include patient preferences, comorbid conditions, procedural findings, opinion of the primary operator (cardiologist who performed the elective diagnostic angiogram), and hospital culture.^17^, ^18^, ^19^ With multiple factors driving revascularization decisions, community data show a 2‐fold variation in revascularization attributable primarily to patient factors and procedural findings.^17^

In our analysis of the largest integrated healthcare system in the United States, we found a 1.5‐fold variability between hospitals in the rate of revascularization in the 30 days after a new diagnosis of obstructive CAD by elective angiography. Across all hospitals, 42% of patients were treated with medical therapy alone after diagnosis. We found no major clinical differences in patient demographics and comorbidities between VA hospitals more and less likely to pursue revascularization. Consistent with prior data^17^ and a known mortality benefit of revascularization in certain populations,^20^ we found the rates of proximal LAD, 3‐vessel CAD, and left main obstructive disease were higher in hospitals more likely to pursue revascularization.

Variation in facility‐level revascularization in our study was driven primarily by PCI. Outside of acute presentations of CAD or the diagnosis of 3‐vessel, left main, or proximal LAD coronary disease that confers a mortality benefit with revascularization, current guidelines suggest reservation of revascularization for medicallyre fractory, stable, ischemic heart disease.^21^ Accordingly, the complex decision to pursue revascularization by PCI for nonsurgical CAD is dependent on the physician, patient preferences, patient symptoms, and angiographic findings.^22^, ^23^, ^24^ In addition to varying opinions on the landmark COURAGE trial,^25^ prior work shows cardiologists may overemphasize the benefits of PCI in management of stable obstructive CAD.^26^ It is possible these factors could account for the variability in PCI as an initial treatment after diagnosis of obstructive CAD, noted in our study.

To determine if use of revascularization was indicative of higher overall procedural use or if a specific procedure has a distinct facility‐level pattern of use, we analyzed revascularization and 2‐year post‐PCI stress test rates for each facility. Stress testing in the 2 years following PCI is rarely appropriate outside of new or progressive symptoms.^27^ We found no correlation between the hospital‐level rate of revascularization for CAD and the 2‐year rate of stress testing after PCI. Prior data have shown significant variability within the VA system in 2‐year post‐PCI stress test rates without translation of higher rates of stress test use to improved mortality.^7^ These findings indicate factors influencing facilities to pursue revascularization may be independent of those leading to use of stress tests after PCI. The lack of correlation may also be a result of different physician groups driving these practices (eg, cardiologists driving initial revascularization, and primary care physicians affecting use of post‐PCI stress testing). This lack of correlation suggests methods to reduce variation in use may be more effective if directed toward drivers of a particular procedure at a facility rather than facilitywide.

Our work suggests significant practice variation in the treatment approach to elective diagnoses of obstructive CAD within the VA system. It is unknown how this variability compares with non‐VA healthcare networks. Certain aspects unique to VA‐based care, such as lack of financial incentives for volume of care and resource limitations, may affect the variability noted. We note variation in the approach to obstructive CAD in the absence of financial drivers, highlighting the importance of understanding factors driving this variability (eg, local culture, process of care, and resource availability) that may inform opportunities to achieve more consistent effective care.

Our findings should be considered in light of certain limitations. First, the VA CART system does not record patient symptoms but does capture indication for procedure. However, lack of details on patient symptoms hinders our ability to analyze their impact on patient selection for diagnostic angiography and choice of revascularization strategy. However, systems are being developed to capture patient‐reported health status among patients undergoing elective coronary procedures at the VA.^28^ Second, we cannot account for veterans moving within the VA healthcare system that could affect hospital‐level rates of procedures evaluated in this work. Third, despite robust observational data, there is a possibility of residual confounding that could affect patient selection for revascularization versus medical treatment. To our knowledge, there has been scant prior work describing facility‐level variation in revascularization versus medical therapy to a new diagnosis of obstructive CAD. In addition, we chose not to evaluate stress testing after CABG because of the extended window of follow‐up in which stress testing is considered rarely appropriate (within 5 years of surgery) as this would have significantly limited the cohort with adequate follow‐up. Despite the limitations above, our work addresses this knowledge gap through analysis of the largest integrated healthcare system in the United States.

## Conclusions

In this national registry of the largest integrated healthcare system in the United States, the median facility‐level rate of revascularization was 59.6%, with large variability between hospitals, from 41.5% to 88.1%, primarily driven by the rate of PCI. Variation in facility‐level revascularization was primarily because of PCI rather than CABG. Facility rates of revascularization were not associated with use of stress testing, suggesting use of individual procedures may not reflect measures of use for other procedures. Further study is needed to define factors driving variation noted in our work to promote effective and efficient care.

## Disclosures

Dr Sandhu had full access to all of the data in the study and takes full responsibility for the integrity of the data and the accuracy of the data analysis. The views expressed in this article are those of the authors and do not necessarily reflect the position or policy of the Department of Veterans Affairs or the US government..
